# Ectopic germinal centers in the nasal turbinates contribute to B cell immunity to intranasal viral infection and vaccination

**DOI:** 10.1073/pnas.2421724122

**Published:** 2025-03-20

**Authors:** Romain Gailleton, Nimitha R. Mathew, Laura Reusch, Karin Schön, Lydia Scharf, Anneli Strömberg, Andrea Cvjetkovic, Luaay Aziz, Johan Hellgren, Ka-Wei Tang, Mats Bemark, Davide Angeletti

**Affiliations:** ^a^Department of Microbiology and Immunology, Institute of Biomedicine, University of Gothenburg, Gothenburg 413 90, Sweden; ^b^Department of Clinical Immunology and Transfusion Medicine, Region Västra Götaland, Sahlgrenska University Hospital, Gothenburg 413 46, Sweden; ^c^Department of Otorhinolaryngology, Head & Neck Surgery, Region Västra Götaland, Sahlgrenska University Hospital, Gothenburg 413 45, Sweden; ^d^Department of Otorhinolaryngology, Institute of Clinical Sciences, The Sahlgrenska Academy, University of Gothenburg, Gothenburg 413 90, Sweden; ^e^Department of Infectious Diseases, Institute of Biomedicine, University of Gothenburg, Gothenburg 413 90, Sweden; ^f^Department of Clinical Microbiology, Region Västra Götaland, Sahlgrenska University Hospital, Gothenburg 413 46, Sweden; ^g^Department of Translational Medicine—Human Immunology, Lund University, Malmö 205 02, Sweden; ^h^SciLifeLab, Institute of Biomedicine, University of Gothenburg, Gothenburg 413 90, Sweden

**Keywords:** influenza A virus, B cell, germinal center, upper respiratory tract, mucosal immunity

## Abstract

The nasal mucosa is the first immunologically active site that respiratory viruses encounter and establishing immunity at the initial point of pathogen contact is essential for preventing viral spread. Influenza A virus (IAV) in humans preferentially replicates in the upper respiratory tract (URT) but mouse models of infection result in lower respiratory tract infection. Here, we optimize IAV inoculation to enhance replication in the nasal turbinate (NT) and study local B cell immunity. We demonstrate that URT-targeted IAV infection stimulates robust local B cell responses, including germinal center (GC) B cell formation in the NT, outside of classical nasal-associated lymphoid tissues. NT GC contributes to local tissue-resident B cell generation and enhances local antibody production. Furthermore, URT-focused immunization also induces significant GC formation in the NT. Finally, we detect steady-state GC in the NT of both mice and healthy humans, suggesting continuous immune surveillance triggered by environmental stimuli. These findings highlight the pivotal role of the NT in local and systemic immunity, with important implications for future mucosal vaccines targeting the upper airways.

## Significance

Respiratory viral diseases are a significant public health threat. The first line of immune defense is within the upper respiratory tract (URT). Despite this fundamental role, critical aspects of humoral immunity within the URT remain unclear. Germinal centers (GC) are anatomical microstructures where B cells increase their affinity for the antigen and differentiate into effector cells. In our study, we find that GC B cell immunity within the URT of mice is not limited to previously described nasal-associated lymphoid tissues but GCs are distributed across the respiratory tract to induce potent and decentralized antiviral immunity. These cells are also present in healthy adult individuals. Our findings can open up targeting strategies for inhaled mucosal vaccines.

Establishing protective immunity at the first site of viral contact is crucial to prevent infections from spreading (1). The airways, as a mucosal site, include both inductive and effector regions where antigens are processed and trigger an appropriate immune response (2–4). Despite having garnered attention for the potential development of mucosal vaccines (5–7), our immunological understanding of the upper respiratory tract (URT) remains incomplete.

The URT contains key inductive sites, nasal-associated lymphoid tissues (NALT) in mice or the tonsils in humans, composed of M cells, antigen-presenting cells, and adaptive immune cells (8–11). Mucosal sites in the URT may be broadly divided into organized and diffused NALT (O-NALT and D-NALT, respectively) (12, 13). O-NALT is commonly referred to as NALT and is localized just above the palate, while D-NALT are mucosal sites spread within the nasal turbinates (NT). In this study, we will refer to NALT when discussing the O-NALT and NT when discussing the D-NALT. Overall, the presence of inductive sites, outside NALT but within the URT has not been explored.

Influenza A virus (IAV) remains a significant threat to global health, with an annual average of 400,000 deaths worldwide (14). IAV, like other respiratory viruses, enters the body via the respiratory mucosa. In humans, mild infections are self-limited to the URT and viral replication in the lower respiratory tract (LRT) and lung damage is only observed in severe cases. However, murine infection models exhibit almost exclusively LRT viral replication (15, 16) and have not been optimal for studying URT immunity.

NT resident memory CD4^+^ and CD8^+^ T cells have been shown to take part in the local viral clearance (17, 18). In addition, IAV-specific antibody-secreting cells (ASCs) have been found in the NT after infection (19). It is commonly believed that the NALT is the major site for the generation of ASCs during URT-challenge (11, 12). However, one study has surprisingly demonstrated that the removal of NALT and proximal cervical lymph nodes (cLN) did not hamper the development of B cell responses in the NT upon IAV infection (20). Despite these findings, the origin of NALT- and cLN-independent IAV-specific cells has remained elusive for almost two decades.

It is well known that, upon external stimuli, germinal centers (GC) may often self-organize within mucosal tissues. These lymphoid structures are well documented in the gut (GALT), the lungs (BALT), and nasopharynx (NALT) (4, 21, 22). Recently, the presence of GC was also reported close to the dura mater, in structures named dura-associated lymphoid tissue (DALT) (23) and in the skin (24), suggesting that many noncanonical sites may host ectopic GCs.

Here, we set out to determine the NALT-independent origin and dynamics of anti-IAV B cell responses in the URT. By employing a URT-restricted infection method, we could more faithfully mimic the infection dynamics observed in humans thereby demonstrating that intranasal infection and immunization trigger a specific GC B cell response in the NT which fuels the local B cell and antibody (Ab) response. Our data highlight the complexity of B cell responses in the respiratory tract and identify local pathways leading to antiviral immunity.

## Results

### URT-Restricted IAV Infection Elicits Robust Viral Replication and B Cell Immunity in the Nasal Tissue.

**Fig. 1.**
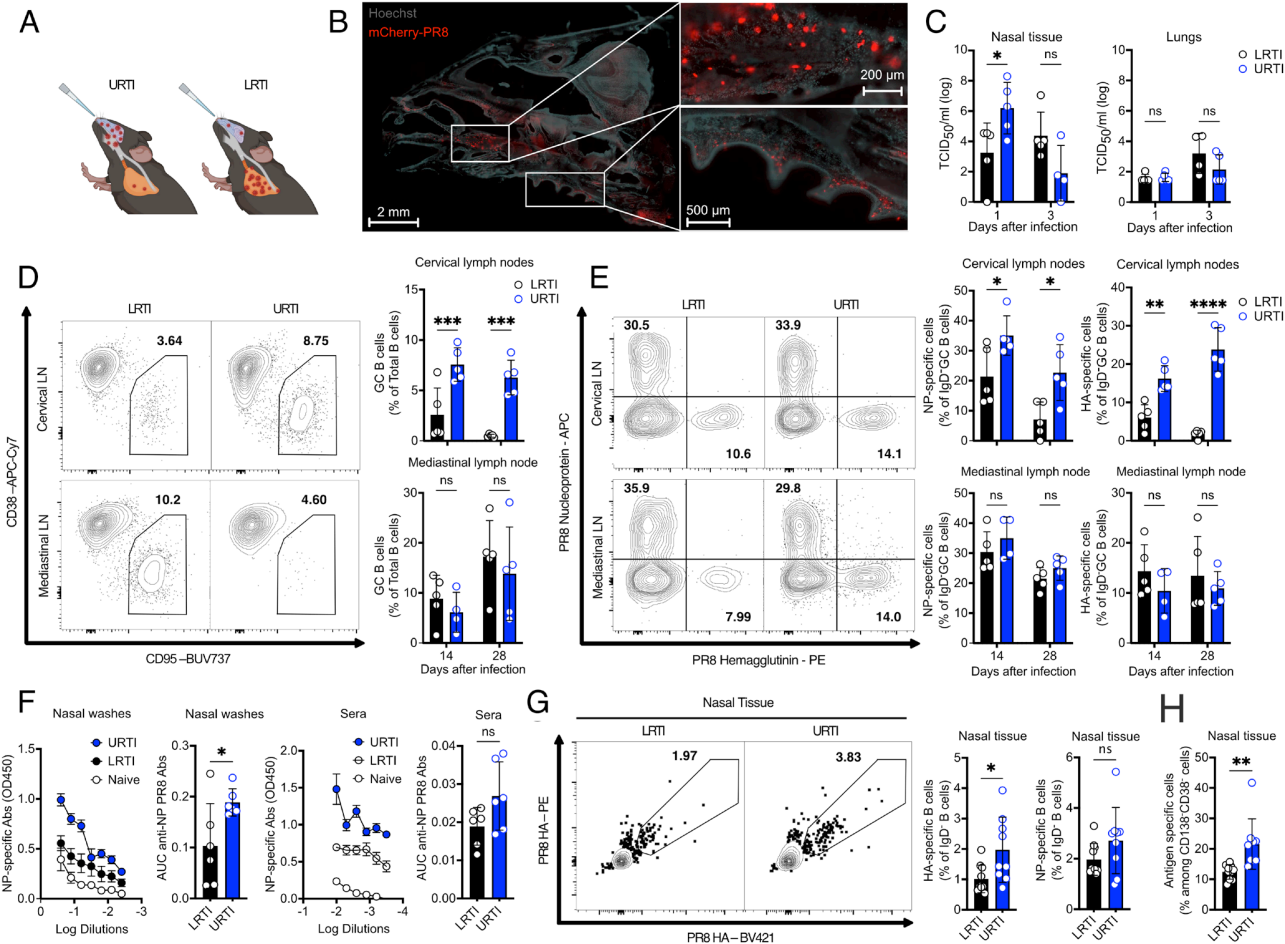
URTI induces robust virus-specific B cell responses within the NT. (*A*) Scheme of the differential method of PR8 infection. URTI consisted in 10 μL of viral inoculum containing 10^5^ TCID_50_. LRTI inoculum contained 500 TCID_50_ diluted in 25 μL. Created with BioRender.com. (*B*) Representative microscopic frontal section of NT stained with Hoechst (light blue) with detection of infected cells after PR8-mCherry (red) infection along the nasal cavities. (*C*) Log TCID_50_ calculation on infected NT and lungs lysates after differential infection methods. Mean ± SEM; one-way ANOVA test. Shown is one representative of three independent experiments (n = 5). (*D* and *E*) Representative flow cytometry plot of total (*D*) or IAV-specific (*E*) GC B cell gating in URTI vs LRTI in mLN and cLN. On the *Right* are represented on bar graphs total (*D*) or IAV-specific (*E*) GC B cells percentages among total B cells after either URTI or LRTI at 14- and 21-d post infection. Mean ± SEM. Shown is one representative of two independent experiments (n = 5); one-way ANOVA test. (*F*) Serial dilution curves of NP-specific Ab from nasal washes (NW) and sera of mice at steady state or 28 d post infection (URTI or LRTI). Mean ± SD; one-way ANOVA test was conducted between URTI and LRTI conditions. AUC were calculated and plotted from detected anti-NP Abs in NW and sera. Mean ± SEM; Student’s *t* test. One representative of three independent experiments (n = 6). (*G*) Representative flow plot of HA-specific B cell gates 28 d post URTI or LRTI. Plotted populations on bars with Mean ± SEM. (*H*) Bar graph representation of antigen-specific CD138^−^CD38^−^ B cells, comparison of both populations following URT or LRT infection methods. Mean ± SEM, (*G* and *H*) shown is one representative of two independent experiments (n = 9). ns *P* >0.05; *P < 0.05; **P < 0.005; ***P < 0.0005.

When adaptive immune responses are studied after intranasal (i.n.) infection with IAV in mice, typically relatively large volumes of inoculum with low viral titers are used (25). With this approach, virions are likely to reach the LRT, resulting in reproducible viral replication in the lungs and robust immune response in lung-draining mediastinal lymph node (mLN) (26, 27). Thus, the method is well suited for studying lung-related symptoms of respiratory infections; however, it is not reflecting the situation in humans well, where most of the viral replication takes place in the URT (28). Therefore, we adapted a previously reported approach (17), according to which the volume of the inoculum is reduced while the viral titer is increased, facilitating viral replication in the lower temperature environment of the NT (Fig. 1 *A* and *B*). To confirm the efficacy of this approach, we infected mice with either infection methods, using fluorescent mCherry-PR8 virus (29). Measurement of live virus via TCID50 assays confirmed a significant increase of replication-competent viruses in the NT at 1 d post infection (d.p.i). after URT-focused infection (URTI) compared to the conventional LRT infection (LRTI) with no differences observed in lungs or at later time points (Fig. 1*C*).

Given that the primary site of viral replication varied depending on the infection method, we hypothesized that the main draining lymph nodes may also change accordingly. Indeed, total and IAV-specific GC B cells showed an increased frequency in the cLN following URTI, while the response remained unchanged in the mLN (Fig. 1 *D* and *E* and *SI Appendix*, Fig. S1*A*). Together, our data suggest the local viral load to be crucial in determining the priming of local LN. Furthermore, previous studies described the NALT as central inductive sites in the URT (11, 30). Similar to cLN, URTI induced a much stronger GC reaction in the NALT, even though the frequency of antigen-specific cells was not significantly different from LRTI (*SI Appendix*, Fig. S1 *B* and *C*).

To determine the overall impact on humoral immunity we evaluated antigen-specific antibodies (Abs) systemically in sera and in NW. Local nasal Abs are pivotal in protecting from viral reinfection (31, 32). Surprisingly, URTI promoted significantly higher levels of anti-NP Abs not only in NW but also in sera, and more anti-Hemagglutinin (HA) Abs in sera (Fig. 1*F* and *SI Appendix*, Fig. S1*D*) demonstrating URTI to be more potent than LRTI in inducing a humoral immunity. This suggests that URTI could induce higher production of Abs or redirect homing of ASC to the URT.

Thereafter, we sought to enumerate HA and NP-specific activated resident B cells (defined as B220^+^ CD19^+^ IgD^−^ and CD45i.v.^−^ cells) in NT to define how local virus-specific B cell compartment was affected by infection mode. NT along with the septum and associated tissues were manually separated from the palate containing the NALT before processing and analysis (*SI Appendix*, Fig. S1*E*). Indeed, URTI resulted in a higher frequency of IAV-specific resident B cells within the NT, possibly contributing to the higher concentration of local Abs (Fig. 1*G*). Conversely, intratracheal infection (*SI Appendix*, Fig. S1 *F* and *G*) ensued no IAV-specific B cells in the URT at 14- and 30-d postinfection, indicating that IAV-specific B cells found in the URT originate solely from the cLN or URT itself. Among antigen-specific IgD^−^ B cells, CD38^−^ CD138^−^ IgD^−^, nonnaïve B cells, with a GC-like phenotype, constituted over 20% of IAV-specific B cells within the NT itself and significantly increased after URTI (Fig. 1*H*).

These findings underscore the significance of the infection route in shaping both local and systemic immune responses to IAV. URTI resulted in higher viral titers in the NT that in turn led to enhanced local and systemic humoral immunity. Furthermore, URTI induced a significant increase in antigen-specific GC B cells in the cLN as well as the NALT and influx of B cells in the NT, including a “GC B cell”-like population. The latter observation was of particular interest, given that the NALT were removed prior to analysis and thus suggested the presence of ectopic lymphoid structures within the NT itself.

### NT GCs Are Implicated in IAV-Specific B Cell Responses Upon Intranasal Infection.

A recent study demonstrated the crucial role of NALT as inductive site for ASC generation during local immunization with 4-hydroxy-3-nitrophenyl (hapten) (NP-hapten) admixed with potent TLR4 agonist adjuvant (11). There, ASCs were shown to egressed from the NALT and reached the NT via circulation. However, an earlier study reported the presence of IAV-specific Abs in the nasal lumen in infected animals where NALT and cLN were surgically removed, raising the possibility that other nasal sites may be more important during infection (20). In addition, a relatively large volume of immunizing substance was used in the study by Liu et al., possibility also triggering a LRTI-like response (11). The presence of an alternative site for activation is supported by our observation of GC-like cells within the NT itself (Fig. 1*H*) that may be organized in GC-like structures similar to DALT, recently described in the dura mater (23).

**Fig. 2.**
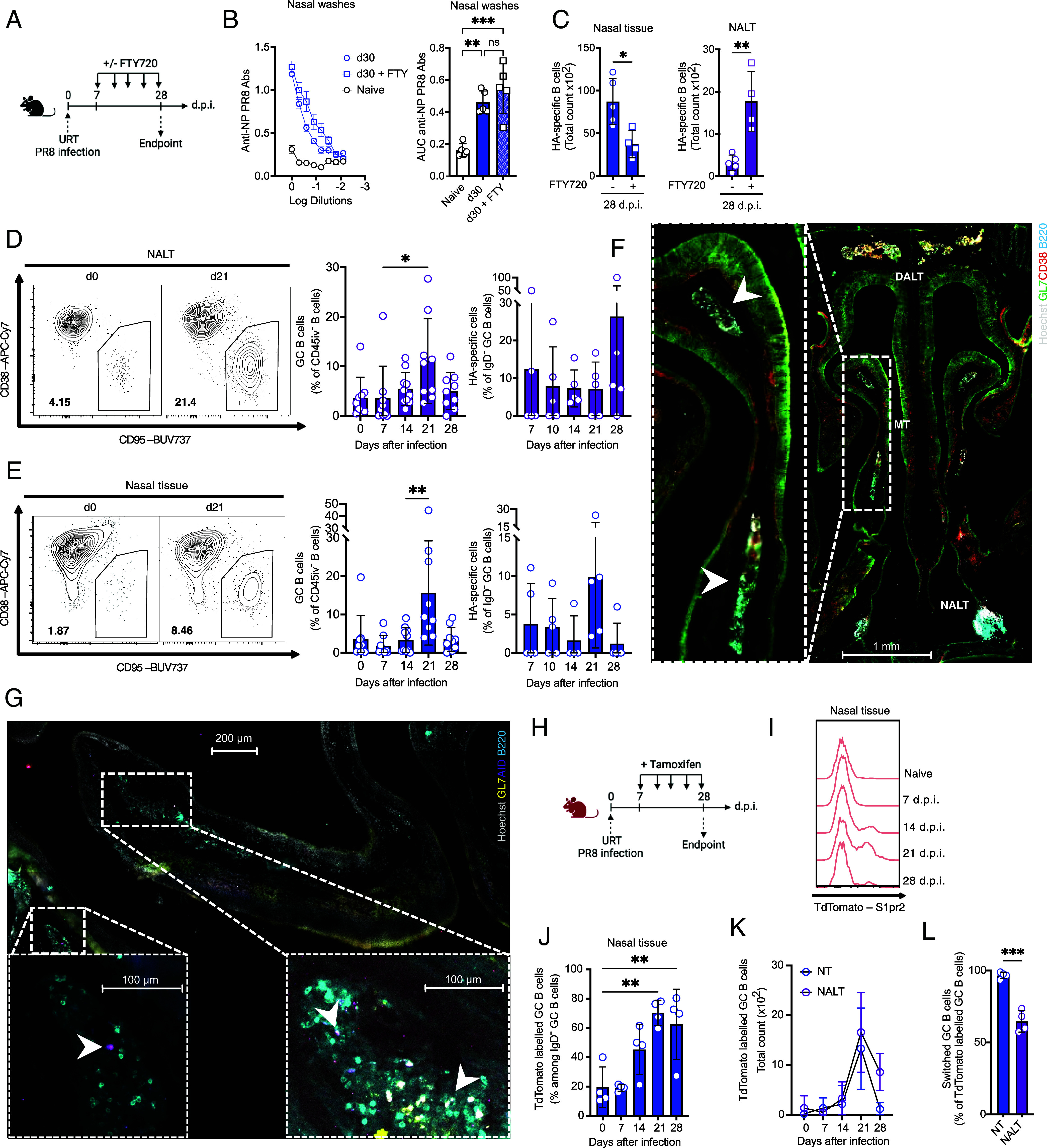
The nasal tissue hosts Influenza-specific germinal center B cells after infection. (*A*) Scheme of experiment strategy. Created with BioRender.com. (*B*) Dilution curves of detected NP-specific Abs in NW from mice at steady-state or 28 d after URTI with or without FTY720 treatment. Mean ± SD; Bar graphs comparison of calculated AUC from above-mentioned groups. Mean ± SEM; one-way ANOVA test. One representative experiment of two independent ones (n = 5). (*C*) Bar graphs of HA-specific NT and NALT CD45iv^−^ IgD^−^ B cells total count 28 d after URTI with or without FTY720 treatment. Mean ± SEM, (n = 5); Student’s *t* test. (*D* and *E*) Representative flow gates of CD45iv^−^ GC B cells at day 0 and 21 post URTI along with bar graphs of total or HA-specific GC B cells temporal dynamic percentages in NT (*D*) and NALT (*E*). Mean ± SEM; (n = 8 and 9), from two independent experiments; one-way ANOVA test. (*F* and *G*) NT frontal (*F*) and sagittal (*G*) microscopy sections of infected mice 21 d post URTI with close-up to the middle turbinates (MD) with indicated staining colors. (*H*) Scheme of experiment strategy with S1pr2-mice. Created with BioRender.com. (*I*) Representative histogram plots of NT TdTomato intensity in CD138^−^IgD^−^ GC B cells at the indicated timepoints post URTI. (*J*) Bar graph temporal dynamic comparison of TdTomato labeled GC B cells percentages in the NT. Mean ± SEM; (n = 4 and 5); one-way ANOVA test. (*K*) Curve plot representation of TdTomato labeled CD138*^−^*IgD^−^ GC B cells total count dynamic in NT, NALT, and lungs at indicated timepoints post URTI. Mean ± SEM; (n = 4 and 5). (*L*) Switched (IgM^−^IgD^−^) B cell proportions among TdTomato labeled CD138^−^IgD^−^ GC B cells from day 21 post URTI. Mean ± SEM; Student’s *t* test (n = 4 and 5). ns *P* > 0.05; *P < 0.05; **P < 0.005; ***P < 0.0005.

Therefore, to determine whether the NT was a bona fide inductive site, we investigated whether the GC-like cells detected in the NT (Fig. 1*H*) arose in situ or from the NALT. We first blocked cellular egress using fingolimod (FTY720) treatment after URTI between 7 and 28 d.p.i. (Fig. 2*A*). This treatment did not impact the magnitude of NP nor HA-specific Abs in the NWs (Fig. 2*B* and *SI Appendix*, Fig. S2*A*). Similarly, while antigen-specific B cells accumulated in the NALT, they were reduced but not completely abrogated from the NT posttreatment, and still in higher number as compared to NALT (Fig. 2*C*). While we can’t definitively prove the effectiveness of FTY720 in blocking NALT to NT transport and vice versa, it is important to note that B cells indeed accumulated in NALT and reduced in NT, and that egress from NALT takes place via blood circulation (11). Furthermore, it is critical to consider that FTY720 may also block the influx of naïve cells and effector cells from lymphoid organs. The data suggest an important but not indispensable role of the NALT in mounting a virus specific-cellular response in the NT. Interestingly, M cells with an antigen-sampling role have been identified in the upper airways that are outside of NALT (33). Indeed, we hypothesize a balance between inoculum dose, antigen localization and availability, and strength of signals to determine the inductive site after i.n. infection.

Our data point toward a role for local GC-like reaction within the NT. Therefore, we wondered whether GC existed within the NT and, if so, were specific for the infection. We first carefully measured the presence GC-like B cells longitudinally after URTI in NT *vs* the well-established NALT. The GC response in the NALT followed the expected dynamic (34), with a peak at day 21 (Fig. 2*D* and *SI Appendix*, Fig. S2*B*). Frequency of HA-specific cells, within the GC, varied between mice but remained on average stable (Fig. 2*D*). Intriguingly, the presence of GC B cell in NT followed the same pattern suggesting that the response was triggered by the infection (Fig. 2*E*). Corroborating the specificity of the response was that NT GC clearly harbored IAV-specific cells (Fig. 2*E*). Immunofluorescence analysis of the URT revealed GC B cell niches located at multiple sites within the NT, including the previously identified NALT and DALT (23) (Fig. 2*F* and *SI Appendix*, Fig. S2*C*). Other prominent sites were observed beneath the lamina propria of the NT, in close proximity to the epithelium (Fig. 2*F* and *SI Appendix*, Fig. S2*D*), an area positioned in close proximity to M cells (33). Following IAV infection, ordered structures with an accumulation of AID-expressing B cells were observed in these areas, supporting the idea that the core of the NT may function as an ectopic GC niche, by facilitating the induction of specific B cells in response to infection (Fig. 2*G* and *SI Appendix*, Fig. S2 *D* and *F*). To further substantiate the specificity and dynamics of GC-derived B cells in the airways post IAV-infection, we infected *S1pr2-CreERT2-tdTomato* mice (S1pr2-mice) (35) and administered tamoxifen every other day, starting from 7 d.p.i., to label GC B cells and their progeny (Fig. 2*H*). The occurrence of tdTomato-labeled GC B cells in NT showed a similar dynamic as the one we found for antigen-specific GC B cells and were also comparable between NT and NALT (Fig. 2 *I* and *J* and K). Notably, when looking at class switching at 21 d.p.i., almost all tdTomato^+^ cells within the NT GC were class switched while a relatively large proportion of tdTomato^+^ NALT GC B cells remained IgM^+^ (Fig. 2*L*), indicating a stronger pressure to class switch in the NT and that the functional role of GC-derived B cells may vary depending on their location within the airway. Nevertheless, proportion of IgA^+^ tdTomato^+^ GC B cells was similar between anatomical sites (*SI Appendix*, Fig. S2*E*).

Furthermore, T follicular helper cells (Tfh) are crucial for proper GC establishment and selection: we previously described their presence within the NT by single-cell RNA sequencing (18) and here we confirmed their existence in NT by flow cytometry (*SI Appendix*, Fig. S2*G*). In addition, we investigated the dynamic of stromal cells such as follicular dendritic cells (FDCs), expressing CD35, that are indispensable to form efficient GC (36). The frequency of CD35^+^ FDCs in the vicinity of B cells indeed significantly increased in the NT at 21 d.p.i., coinciding with the GC peak (*SI Appendix*, Fig. S2 *H*–*J*). We further validated FDCs’ identity by demonstrating their expression of the specific marker FDC-M1 through microscopy analysis (*SI Appendix*, Fig. S2*J*).

### NT GC Exhibit Canonical Features, Including Somatic Hypermutation and Light Zone Dark Zone Segregation.

**Fig. 3.**
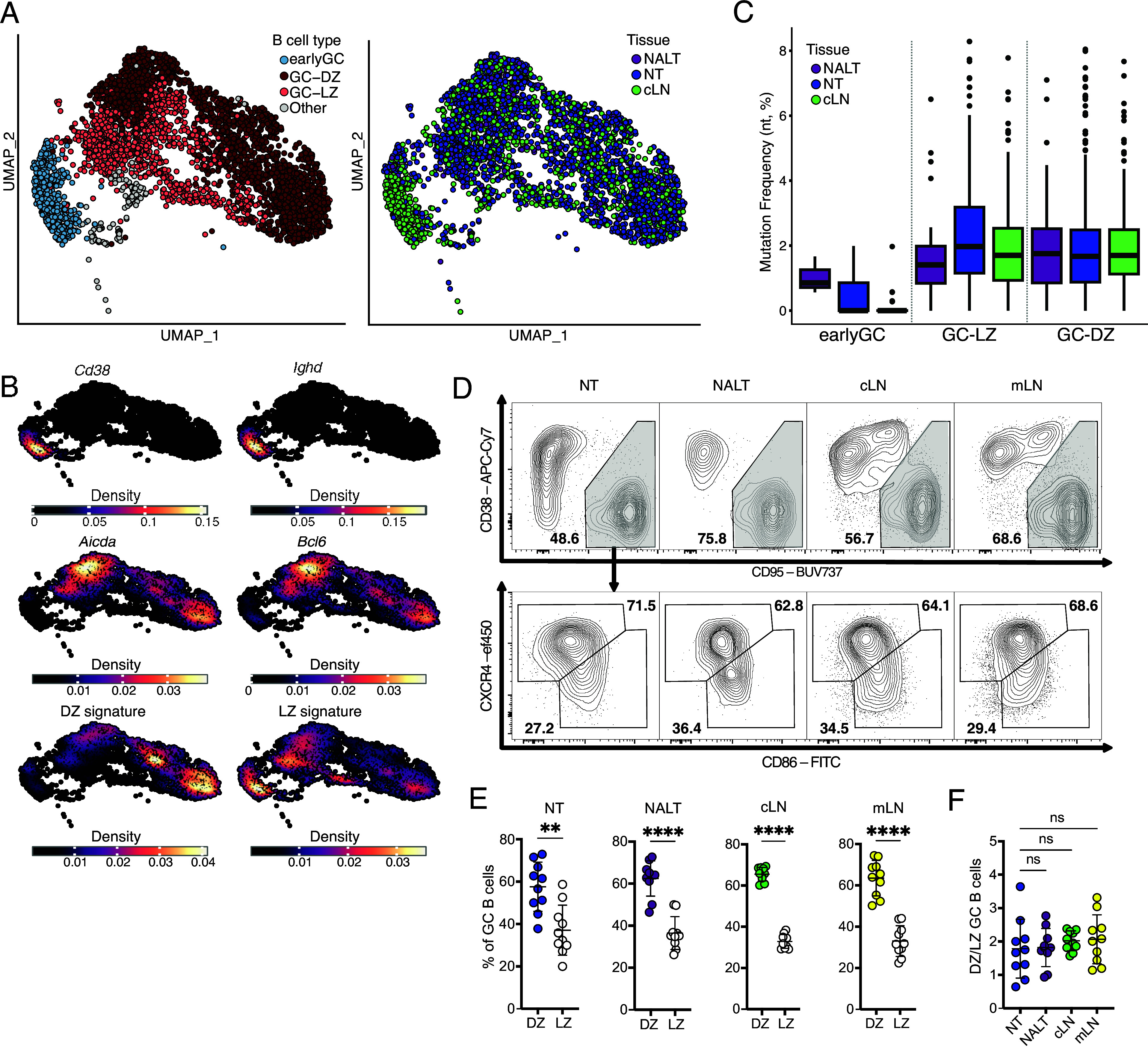
Germinal center B cells in NT exhibit canonical features. (*A*) UMAP visualization of single-cell RNA sequencing data from sorted TdTomato^+^ GC B cells in the NT, cLN, and NALT. Cells are grouped either based on cell types according to signatures identified in B or to organ of origin. (*B*) Density Plot showing gene expression of *Cd38*, *Ighd*, *Aicda*, *Bcl6,* and DZ and LZ signatures (37) on the UMAP. (*C*) Box plot showing the BCR nucleotide mutation frequency among B cell types, as identified in *A*, across tissues (NT, blue; NALT, violet; cLN, green). Data are presented as median and interquartile range. (*D*) Representative flow cytometry plot of gated GC B cells and their division into DZ and LZ GC B cells, based on CXCR4 and CD86 expression, across NT, NALT, cLN, and mLN. (*E*) Dot plot comparing the frequencies of DZ and LZ GC B cells across NT, NALT, cLN, and mLN. Mean ± SD. Statistical analysis performed using a *t* test. Data represent two independent experiments (n = 10). (*F*) Dot plot displaying the ratio of DZ to LZ GC B cells in NT, NALT, cLN, and mLN. Mean ± SD. Statistical analysis performed using one-way ANOVA. Data represent two independent experiments (n = 10).

Our data so far are consistent with the presence of cells in the NT that have phenotypical features consistent with a GC phenotype. To definitively establish the GC B cell identity of the cells, we infected S1pr2-mice and treated them with tamoxifen daily until 21 post infection. TdTomato^+^ GC B cells from the NT, NALT, and cLN were sorted and subjected to single-cell RNA and BCR sequencing. UMAP clustering revealed several populations with the majority of these being GC B cells, as expected (Fig. 3*A*). In details, we could define a cluster of “early-GC” B cells, mostly present in cLN, and we could divide GC B cells into dark zone (DZ) and light zone (LZ), according to well established signatures (37), the distribution of which was almost identical across tissues (Fig. 3 *A*-*B* and *SI Appendix*, Fig. S3*A*). The presence of these distinct phenotypes within the GC reaction reflects the organized and functional structure of GCs. Other B cells were mostly IgM and were only a minor fraction across tissues (Fig. 3*A* and *SI Appendix*, Fig. S3 *A*-*B*). Expression of key GC-associated genes, including *Aicda, Bcl6*, *Fas*, *Mki67, Gcsam, S1pr2, Icosl,* and *Myc* was strong in the GC clusters and shared across tissues, thus confirming the bona fide GC transcriptional signature of NT GC B cells (Fig. 3*B* and *SI Appendix*, Fig. S3*C*). Nevertheless, when performing differential gene expression (DEG) analysis between NT *vs* NALT (*SI Appendix*, Fig. S3*D* and Dataset S1) and NT vs. cLN (*SI Appendix*, Fig. S3*E* and Dataset S1), in the individual clusters, we could identify several DEG, consistent with the unique, ectopic nature of NT GC B cells. For instance, *Tgif1*, a TGFβ induced factor, was consistently upregulated in NT. In addition, GC B cells from NT, cLN, and NALT, but not early GC-like B cells, demonstrated signs of somatic hypermutation, also depending on Ig class expression, as expected (Fig. 3*C* and *SI Appendix*, Fig. S3*F*). Notably, we could not find any IgA-expressing B cell within the cLN (*SI Appendix*, Fig. S3*B*), further strengthening local origin of NT GC. Finally, when looking at clonal expansion and sharing we, again, noticed a completely distinct pattern between organs where most of the clonally expanded B cells were within the NT but not the NALT and cLN (*SI Appendix*, Fig. S3*G*) and, while some clonally related cells were observed between NALT and NT, the majority was unique for their specific GCs (*SI Appendix*, Fig. S3*H*).

To confirm LZ/DZ division, we performed a flow cytometry staining. DZ GC B cells are characterized by the expression of CXCR4^+^CD86^−^ (38) while LZ GC B cells are marked by CXCR4^−^CD86^+^ (39, 40). Indeed, we confirmed that GCs within the NT, NALT, cLN, and mLN exhibit similar proportions of DZ and LZ GC B cells (Fig. 3 *D*–*F*), with a higher percentage of cells in the DZ, consistent with the proliferative phase of GC B cells. This suggests that GC dynamics in the NT closely mirror those observed in other lymphoid tissues, reinforcing the functional role of NT GCs in B cell development.

In summary, our findings demonstrate the presence and inductive role of NT GC during the B cell response following IAV infection. While NALT plays an important role in generating a specific humoral response in the upper airway, they do not appear essential for the development of an infection-specific B cell compartment within the NT. The scRNA and BCR sequencing data further validated that NT GCs develop independently of NALT. Our results, alongside recent studies showing GC responses in DALT (23), emphasize the complexity of the induction of B cell–mediated immunity within the URT.

### URT-Restricted Immunization Triggers Nasal Tissue Germinal Center Formation.

**Fig. 4.**
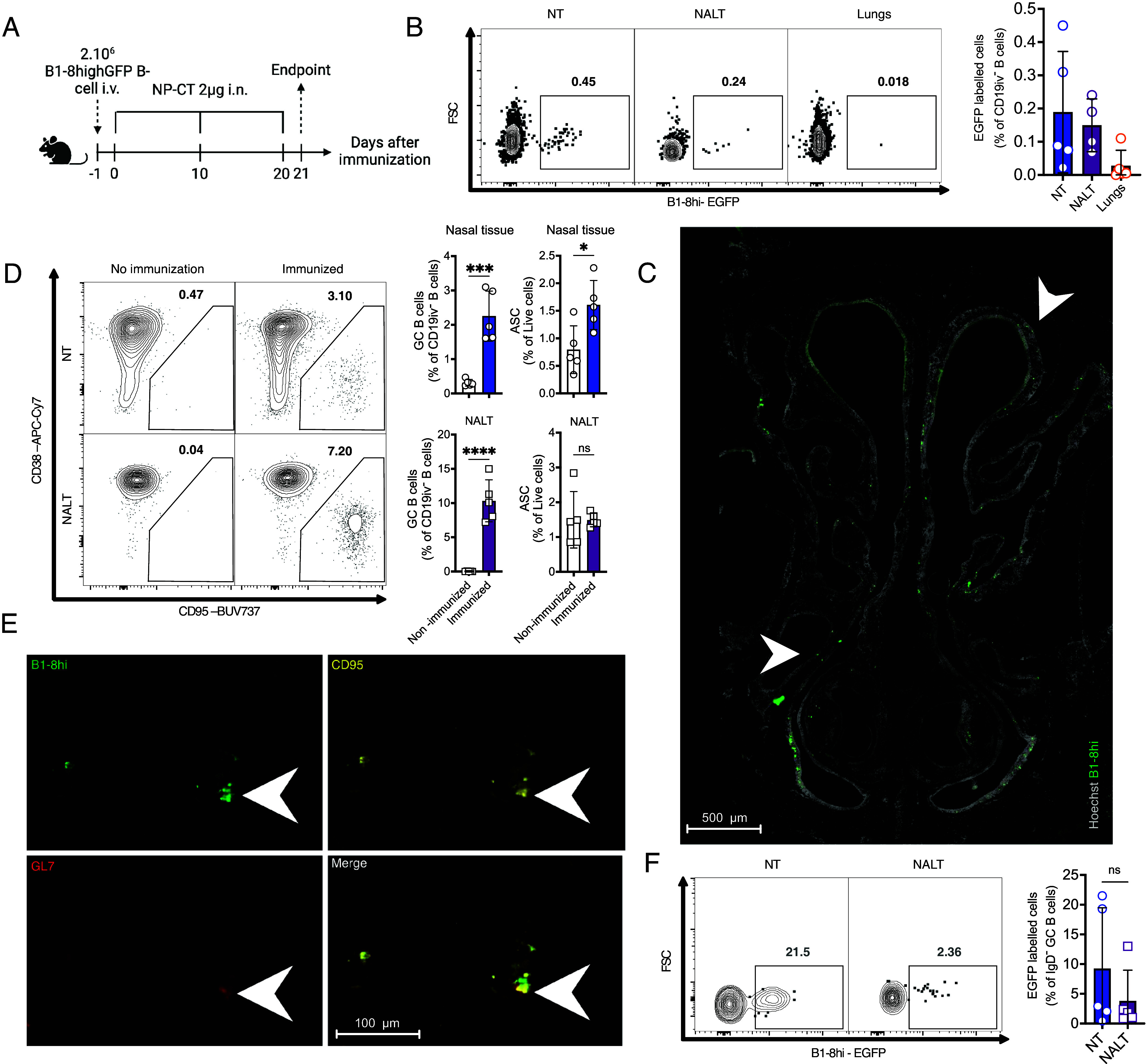
URT-restricted immunizations promote GC B1-8^hi^ cell responses in the NT. (*A*) Scheme of experiment strategy. Created with BioRender.com. (*B*) Representative flow plots of NT, NALT, and lungs EGFP^+^ cells among CD19iv^−^ B cells along with their percentages plotted on bars. Mean ± SEM; (n = 5). (*C*) NT frontal section of a mouse immunized with NP-CT after adoptive transfer with B1-8^hi^ cells as indicated in Fig. 3*A*. (*D*) Representative flow gates for GC B cells gated from CD19iv^−^ B cells in NT and NALT with or without immunization. GC B cells and ASC percentages are plotted per organs and conditions and differences are evaluated between immunized and nonimmunized groups. Mean ± SEM; (n = 5), unpaired multiple *t* tests. (*E*) Microscopic NT frontal section of a mouse immunized with NP-CT in a URT-restricted manner after adoptive transfer with B1-8^hi^ cells showing CD95^+^GL7^+^ B1-8^hi^ cells in NT niches. Quadrants represent single color positive staining and merge duplicates of a close-up on a B cell cluster of the NT. (*F*) Representative flow gates of B1-8^hi^ labeled GC B cells populations in NT and NALT (*Left*) and the quantification in percentages among CD19iv^−^IgD^−^GC B cells (*Right*). Mean ± SEM; (n = 5). ns *P* >0.05; *P < 0.05; **P < 0.005; ***P < 0.0005. ****P < 0.00005.

To further our findings and dissect the role of NT GC upon i.n. immunization we took advantage of the potent cholera toxin adjuvant (CT) conjugated with the small NP-hapten (NP-CT) (41). We transferred 2 × 10^6^ high-affinity IgD^−^ hapten-specific splenic B1-8^hi^-GFP B cells i.v. to C57BL/6J that were subsequently immunized i.n. thrice with 2 µg of NP-CT in 10 µL at 10 d intervals (Fig. 4*A*). We used low volume immunization to mimic the URTI and allow for a local response. NT, NALT, and lungs were collected 24 h after the final immunization. The immunized group showed clear signs of expansion of specific cells in the URT, including NT, but the cells were almost absent in the lungs (Fig. 4*B*). EGFP-labeled cells were found on most of the NT surface (Fig. 4*C*) as previously described after immunization with NP-OVA adjuvanted with a TLR4 ligand (11). In addition, GC B cells could be readily detected in the URT of the immunized group by flow and by microscopy in niches similar to the ones hosting tdTomato-labeled GC B cells, after infection (Fig. 4 *D* and *E* and *SI Appendix*, Fig. S4*A*). GC B cells in the NT and NALT showed frequencies of EGFP^+^ cells that indicated a potent stimulation of all URT inductive sites after immunization with CT (Fig. 4*F*).

Our data again underline the capability for the NT to induce antigen-specific ectopic GC reaction independent from those in NALT and other sites also upon repeated local immunization. Previous findings demonstrating the role of the NALT in the generation of the local ASC response (11) and our current findings support the view that both NALT and NT GC B cells contribute to upper airways B cell immunity, at least when triggered with strongly stimulatory signals.

### Germinal Centers Are Present Within the Nasal Tissue of Healthy Individuals.

Antigen-driven GC constitutively initiate in the digestive tract specific-pathogen-free (SPF) mice while they are almost nonexistent in germ-free mice. This indicates that the microbiome at the interface between the mucosa and the environment to be the main sources of antigens fueling the steady-state GCs (23, 42, 43). Interestingly, we found that the NT was also capable of hosting challenge-unspecific GC B cells (Fig. 2*E*; see day 0).

To further investigate steady-state GC reactions happening in the NT, we treated naïve, uninfected S1pr2*-*mice with tamoxifen and with or without FTY720 for a short time (5 d) before harvesting the URT organs (*SI Appendix*, Fig. S4 *B* and *C*). Spleens were harvested as a source of GC reactions at steady-state (44). All organs hosted TdTomato^+^ cells at this early stage after treatment, regardless of FTY720 administration. However, while the proportion of GC-derived (TdTomato^+^) B cells in the NALT and spleen remained similar between the FTY720-treated and untreated groups, NT GC-derived B cells were significantly more abundant in the FTY720-treated group. This finding suggests that steady-state NT GC-derived B cells do not rely on migration from distal organs but rather originate within the NT itself and potentially migrate to peripheral tissues (*SI Appendix*, Fig. S4 *D* and *E*). These data support the hypothesis that steady-state GC B cells can arise locally within the NT. Along these lines, TdTomato-labeled GC-derived B cells were found across all organs at steady state after 28 d of tamoxifen gavages (*SI Appendix*, Fig. S4 *F*–*H*), with the highest presence in the NALT.

**Fig. 5.**
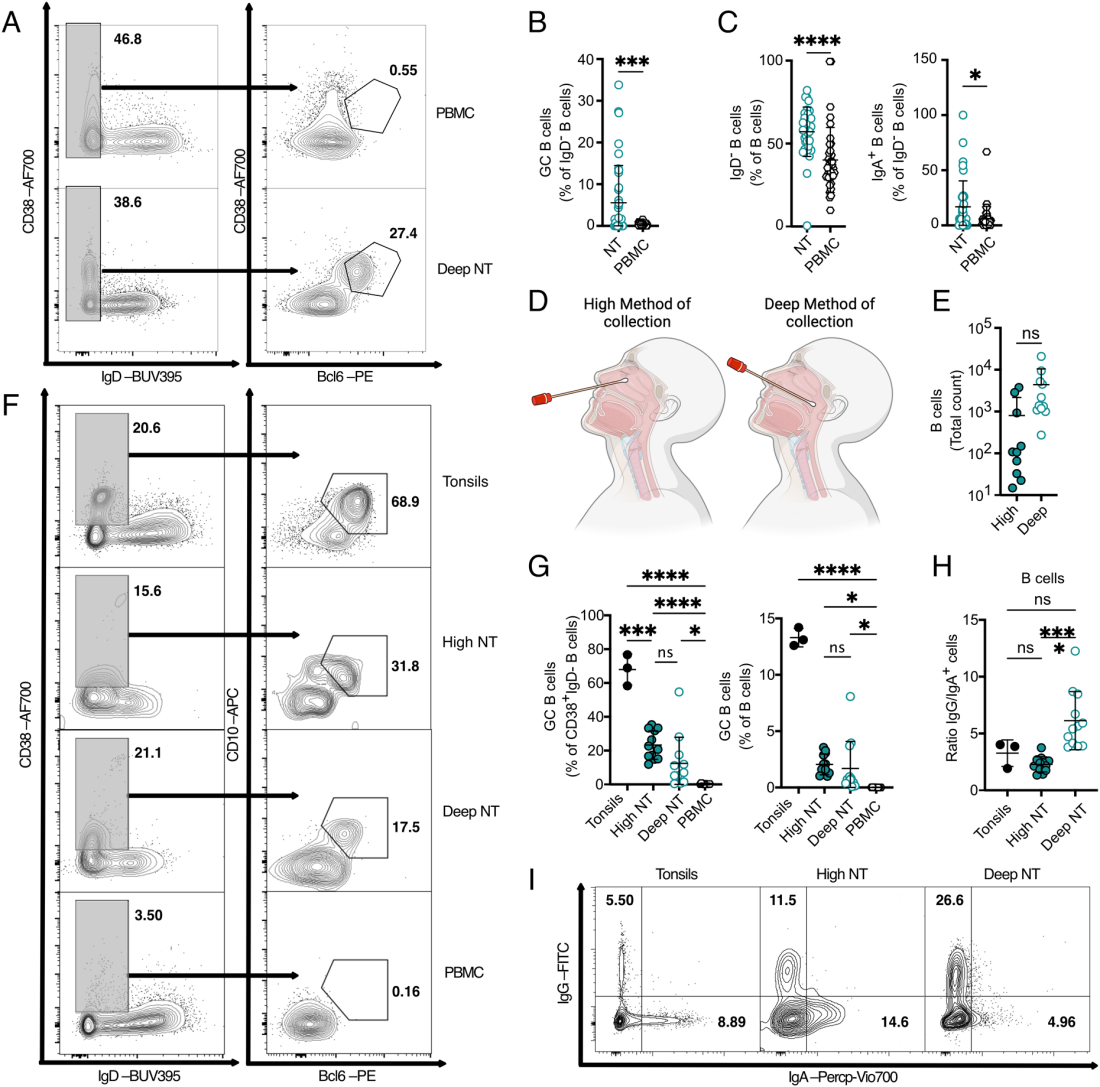
Human nasal tissue retains germinal center reaction in the absence of detectable active infection or challenge. (*A*) Representative flow cytometry plot showing the gating strategy for GC B cells from human nasopharyngeal swabs collected from deep nasal cavity tissue (Deep NT) and peripheral blood mononuclear cells (PBMC) as a negative control. (*B*) GC B cell frequencies among IgD^−^ B cells displayed as dot plots, comparing human Deep NT and PBMC. Data is from three combined independent experiments. Mean ± SD. Statistical analysis performed using a *t* test (n = 36). (*C*) (*Left*) Frequencies of IgD^−^ B cells among total B cells, plotted as dot plots and detected in human Deep NT and PBMC. (*Right*) Frequencies of IgA^+^ B cells among IgD^−^ B cells, also plotted as dot plots, in Deep NT and PBMC. Data is from three combined independent experiments. Mean ± SD. Statistical analysis performed using a *t* test (n = 36). (*D*) Schematic representation of human nasal swab collection methods, illustrating the collection from Deep NT and higher nasal cavities (High NT). Created with BioRender.com. (*E*) Dot plot comparison of the total count of B cells collected using the high and deep nasal swab methods in healthy individuals. Data represent one of four independent experiments. Mean ± SD. Statistical analysis performed using a *t* test (n = 12). (*F*) Representative flow cytometry plot showing the gating strategy for human GC B cells based on CD10 expression from tonsils (positive control), Deep NT, High NT, and PBMC (negative control). (*G*) GC B cell frequencies among CD38^+^ IgD^−^ B cells (*Top*) and among total B cells (*Bottom*), shown as dot plots for human tonsils, High NT, Deep NT, and PBMC. Data represent two independent experiments. Mean ± SD. Statistical analysis performed using a one-way ANOVA test (3 < n < 12). (*H*) Ratio of IgG^+^ to IgA^+^ B cells displayed in dot plots, comparing tonsils, High NT, and Deep NT. Data represent two independent experiments. Mean ± SD. Statistical analysis performed using a one-way ANOVA test (3 < n < 12). (*I*) Representative flow cytometry plot showing the gating strategy for IgA^+^ and IgG^+^ B cells from human tonsils and nasopharyngeal swabs collected from Deep and High NT.

Spontaneous, chronic GC are present and have been extensively studied at gut mucosal surfaces (4) but also during autoimmune diseases (45, 46). In light of our findings, we hypothesized that healthy human NT may host similar responses. To explore this, we collected nasal swabs from healthy volunteers and assessed the presence of GC B cells, characterized by CD38^+^ Bcl6^+^ expression (Fig. 5 *A* and *B* and *SI Appendix*, Fig. S5*B*). The B cell population in the NT displayed a lower proportion of naïve B cells compared to peripheral circulation, along with a higher proportion of IgA^+^ B cells (Fig. 5*C*) This is consistent with the possibility that our swab samples may have included cells from the adenoids (human NALT), as highlighted in a recent study (47).

Building upon this, we carefully collected two types of nasal swabs from different donors: one targeting the superior turbinate (High swab) and the other directed toward the back turbinates and adenoids (Deep swab). No significant differences in the number of B cells recovered from either swab type were observed (Fig. 5 *D* and *E*). The expression of the residency marker CD69 (48, 49) (*SI Appendix*, Fig. S5*C*) and the differentiation marker CXCR3 (50) (*SI Appendix*, Fig. S5*D*) were decreased in GC B cells, indicating their migratory and undifferentiated state. To further confirm the GC B cell identity and increase the specificity we added the commonly used marker CD10. Now, we defined GC B cells as CD19^+^ CD20^+^ IgD^−^ CD38^+^ BCL6^+^ CD10^+^ and compared the percentages and numbers of GC B cells using tonsils as positive controls and PBMC as negative controls (Fig. 5*F* and *SI Appendix*, Fig. S5*A*). Interestingly, the High NT samples, taken from regions distant from the conventional human NALT of the Waldeyer’s ring, displayed a phenotype consistent with GC B cells in most individuals, suggesting the presence of ectopic GC B cells in the NT at steady state (Fig. 5*G*). Unexpectedly, the numbers of GC B cells in both High and Deep NT regions were comparable (*SI Appendix*, Fig. S5*E*), pointing to a significant contribution of ectopic GC B cells in the NT to the steady-state production of locally differentiated B cells. Consistent with previous work (47), isotype expression differed between tonsils, High NT, and Deep NT near the adenoid. Deep NT B cells harbored significantly more IgG^+^ B cells compared to IgA^+^ B cells, whereas Tonsils and high NT had lower IgG/IgA ratio (Fig. 5 *H* and *I*). This confirmed that we sampled different regions and suggested that regional microenvironmental factors may influence class switching or migration patterns of B cells post activation, potentially reflecting distinct functional roles of B cell populations based on their location within the NT. Indeed, as opposed to SPF mice, humans are constantly exposed to environmental challenges that could trigger local GC reactions and induce an IgA-biased response as previously demonstrated in healthy humans (51).

## Discussion

Our findings shed light on the complexity of B cell–mediated immunity in the URT and challenge our current understanding of inductive sites for mucosal immune responses. By employing URTI we were able to more faithfully replicate the human scenario of IAV infection and reveal stromal and immune cells organization in the NT which support a robust local immune response to acute infection.

Our finding, together with the recent identification of NT-resident CD4 and CD8 T cells (17, 18), suggests a prime role for the local immune response in controlling IAV infection at its point of entry, potentially limiting the spread to the lower respiratory tract and reducing the severity of illness (52, 53). Importantly, our results demonstrate that the NT can function as an inductive site for B cell responses, complementing the role of conventional NALT (12, 54). The presence of GC-like structures in the NT, including CD35^+^ FDCs, Tfh, and antigen-specific B cells, in addition to antigenic sampling by M cells in the turbinates (33), reveals a more decentralized and adaptable immune system in the URT than previously thought. Multiple GC niches within the NT, including areas near the epithelium, imply a sophisticated network of inductive sites that can respond rapidly to local antigenic challenges. These are in addition to the recently identified DALT, that has a role in protecting the CNS parenchyma (23), and the well-known NALT. Importantly, the latter have been implicated in ASC production for homing to NT after vaccination (11). Our study complements these findings by identifying NT GC as a significant source of local mucosal B cell responses upon IAV infection. In contrast to Liu et al. (11), we used a more physiological model with a live viral infection that can replicate within the URT while they utilized a hapten-based vaccine in a large volume, which may preferentially induce a response in the NALT and mLN, as we describe for LRTI. We propose that response may be initiated at different anatomical sites in NT, depending on a fine balance between antigen type, local antigen dose, and draining lymph node. Indeed, previous work defined NALT as dispensable for the accumulation of T cells to NT, production of local Abs and for viral clearance after IAV infection (20) and our data offer a conclusive explanation for that observation. Acting as tertiary lymphoid organs following influenza infection, ectopic GC niches in the NT might serve as rapid inductive site in close vicinity to infection loci. Along those lines, nearby inflammation in the turbinates may play a pivotal role in shaping an appropriate GC response (55). Interestingly, we confirmed transcriptional similarities between NT and cLN/NALT GCs, however, these GCs were not identical: first, several DEG were identified, including many consistent with local activation and mucosal cytokine signaling; second, clonal expansion was markedly different between organs and clonal sharing was extremely limited and only between NALT and NT. Future work should address the contribution of these GC structures to local and systemic immunity, including relative output of memory B cells, ASC, and mucosal Abs.

Furthermore, the observation that URT-restricted infection leads to enhanced local and systemic humoral immunity compared to lower respiratory tract infection, taken together with similar findings in humans (56), underscores the importance of local antigen amount and infection route in focusing B cell immunity and should be taken into consideration when designing novel vaccination strategies targeting the URT.

Finally, the detection of steady state GCs in SPF mice and activated GC B cells in healthy human donors underscores the significance of ongoing GC activity in the nasal mucosa, as a first barrier against bacterial and other environmental stimuli. Our findings in human NT imply that steady-state GCs in the nasal cavities contribute to the ongoing differentiation of B cells, potentially conferring site-specific immune protection. Importantly, we carefully sampled a region that was distinct from the deep NT, which corresponds to the adenoids (47). Our study stands in apparent contrast with Ramirez et al. (47), as they could not identify such cells in their mid-turbinate sampling. However, their mid swab targets different anatomical regions as compared to our high swab and may not be a suitable site for detecting these cells. It is interesting to note that, in gut, GC B cells from Peyer´s patches can exit GC and sample antigen directly from M cells in the subepithelial dome (57): we can speculate that a similar mechanism may be at play here and that we are indeed collecting these GC B cells, sampling antigens from the epithelium.

In summary, the finding of NT GCs underscores the need for a more nuanced understanding of mucosal immune responses and their inductive sites. Targeting GC within the NT may prove itself as a viable vaccination strategy to increase local mucosal immunity. By focusing on the NT, i.n. vaccination approaches could enhance local immunity and provide better protection against pathogens that primarily infect the URT such as IAV or SARS-CoV-2 (58, 59).

## Materials and Methods

### Mice.

Female C57BL/6J mice were purchased from Janvier and S1pr2 (B6-S1pr2CreERT2/TdTomatoflox) were bred in house. For NP-specific B-cell experiments F1 mice were generated by crossing C57BL/6 mice with homozygous B1-8high GFP mice. Mice that were infected with influenza A virus PR8 were housed in Animal Biosafety Level-2 conditions. All mice were maintained under specific pathogen–free conditions at the University of Gothenburg. Age- and sex-matched mice were used in the experiments.

### Human Participants.

Healthy donors, stating that they do not have a genetic or drug-induced T-cell inhibition, or respiratory pathologies (asthma, COPD, allergies, regular nose bleeds, or flu-like symptoms) were included in the study. All participants signed an informed consent form before collection of nasal tissue samples utilizing nasopharyngeal swabs (Copan Cat# 4E074S.A) and approximately 7 to 8 mL of venous blood. Blood specimens were collected in heparin-coated tubes and diluted with PBS before being added to lymphoprep solution (Stemcell Technologies) and centrifuged for clear gradient separation of PBMC. Isolated PBMC were reserved in FACS buffer while the nasal tissue samples were processed. Directly after collection, NT cells were separated from the swabs by placing them in 3 mL of 2% fetal calf serum (FCS) supplemented RPMI 1640 Medium (#22400089; Gibco) containing 1.5 mM DTT and placed at 37 °C for 30 min. Cell segregation from the swabs was helped by mixing the samples every 10 min during incubation. PBMC and NT cells were centrifuged for 8 min at 700×*g* before being resuspended in FACS buffer (HBSS, 1 mM of EDTA with 10% FCS) and stained for flow cytometry analysis.

Human tonsils were obtained from healthy children and adults, aged between 2 and 25 y, undergoing either cold-steel tonsillectomy or radiofrequency tonsillotomy at the Otorhinolaryngological department of Sahlgrenska University Hospital, Sweden. The indications for undergoing surgery were OSDB (obstructive sleep disordered breathing) with tonsil hypertrophy or chronic tonsillitis. After surgical removal of the tonsils, they were immediately placed in saline solution and kept on ice during transportation to the laboratory for processing within 24 h. Tonsils were processed to single-cell suspension following the protocol from Wagar et al. (60).

### Viruses and Infections.

Ten-day-old embryonated chicken eggs were inoculated with 10^5^ TCID_50_ of Influenza A/Puerto Rico/8/34 (PR8) or the same strain expressing mCherry, and viruses were grown for 48 h at 35 °C and 55% humidity with gentle rotation. Eggs were chilled at 4 °C for several hours prior to harvesting allantoic fluid, which was then clarified by centrifugation at 30,000×*g* for 3 h, aliquoted, and stored at 80 °C.

For infection, mice were anesthetized with isoflurane and intranasally instilled with either 500 TCID_50_ in 25 μL or 10^5^ TCID_50_ in 10 μL of influenza A virus PR8 diluted in sterile HBSS+0.1%BSA depending on the method of infection. For intratracheal infection mice were intratracheally inoculated with 1050 TCD_50_ of influenza A virus PR8 diluted in sterile HBSS+0.1%BSA in a total volume of 25 μL.

### Immunizations.

NP hapten-specific GFP-labeled splenic λ-expressing B cells were isolated by depleting non-B cells and IgD^+^ κ-expressing cells using an EasySep Mouse B cell isolation kit (Stem Cell Technologies, Manchester, UK), supplemented with 2 μg of anti-mouse κ-chain biotinylated antibody (BD Biosciences, San Jose, CA). 2 × 10^6^ selected B cells were transferred to sex-matched C57BL/6J mice intravenously. For mucosal immunization a restricted volume of NP-CT (10 μL) was administered intranasally in anesthetized mice. To make the NP-CT solution, cholera toxin (CT) was diluted in distilled water for 48 h then mixed with an equal volume of 0.1 m NaHCO_3_ and 20 equiv. NP-OSu (Biosearch Technologies, Novato, CA) per mole CT. The mixture was incubated for 12 h at 4 °C, transferred into a Slide-A-Lyzer dialysis cassette, and dialyzed against 0.05 M NaHCO_3_, followed by water. The protein concentration was determined using a BCA assay (Thermo Fisher Scientific, Rockford, IL). A total of 2 μg NP-CT was used for each intranasal immunization.

### Treatments.

For tamoxifen administration, 100 μL of a 20 mg/mL solution of tamoxifen (Cat. No. T5648; Sigma-Aldrich) dissolved in corn oil (Cat. No. C8267; Sigma) was administered via oral gavage with a 25-gauge needle at indicated periods. for the indicated periods. Fingolimod (Cayman Chemicals) treatments, 2.5 μg/g of mouse body weight were injected intraperitoneally in 100 μL of deionized water at indicated periods.

### Single-Cell RNA Sequencing.

For single-cell sequencing, cells were extracted from 8 male S1pr2 mice 21 d post URTI and following tamoxifen gavages. NT B cells were further enriched using the EasySep™ Mouse B Cell Isolation Kit (Catalog # 19854) following the manufacturer’s instructions. Single suspensions from cLN and NALT and enriched B cells from the NT were stained with anti-B220-BV785 (RA3-6B2), anti-IgD-AF700 (11-26c.2a), and anti-GL7-FITC (GL7) for 45 min at 4 °C in the dark. TotalSeq™-C0301, -C0302, and -C0303 anti-mouse Hashtag Antibodies (Biolegend) were added for the time of incubation to the suspensions of NT, NALT, and cLN cells, respectively. Live cells were then stained with the LIVE/DEAD™ Fixable Aqua Dead Cell Stain Kit (Catalog # L34966) for 20 min after 2 thorough washes with 2 mL of PBS 1X. Live B220^+^IgD^−^GL7^+^TdTomato^+^ cells from the suspensions were sorted in 1 Eppendorf tube containing 50 μL of FCS. The sorted cell count was 17,500 cells from NT, 2,500 cells from NALT, and 10,000 cells from cLN. Pooled sorted cells from the three organs were then directly centrifuged 5 min at 400rcf and the pellet was collected to be processed for sequencing.

Pooled cells were processed into single cells in a chromium controller (10X Genomics). The single-cell gene expression, BCR VDJ, and cell surface protein (CSP) libraries were prepared using Chromium Next GEM Single Cell 5′ Reagent Kits v2 (Dual Index) kit (PN-1000265), library construction kit (PN-1000190), 5′ Feature Barcode Kit (PN-1000256), Chromium Single Cell Mouse TCR Amplification Kit (PN-1000254), Chromium Next GEM Chip K Single Cell Kit (1000286), Dual Index Kit TT Set A (PN-1000215) and Dual Index Kit TN Set A (PN-1000250) (61).

cDNA libraries were quantified using a Qubit Fluorometer (Invitrogen) and quality assessed using an Agilent Tapestation system. The libraries were sequenced on DNBSEQ-G400RS (MGI Tech) on a PE100 flowcell by Xpress Genomics (Solna, Sweden) according to sequencing instructions provided by 10× Genomics.

### Single-Cell RNA Seq Data Processing.

Raw fastq files were processed through the 10X cellranger pipeline using the count command and default parameters with reference genome GRCm38-mm10.

Raw UMI count matrices generated from the cellranger 10X pipeline were loaded and merged into a single Seurat object (Seurat version 5). Hashtags were normalized by a centered log-ratio (CLR) normalization, singlets retained, and binding assigned using HTODemux function. Cells were further filtered according to <10% mitochondrial counts, >150 and <5,000 unique features and no expression of *Cd3e*, *Cd3g*, *Nkg7*, *Cavin3*, *Clec5a,* and *Chgb*. Cells from different organs were integrated using CCAIntegration and gene counts were normalized and scaled with the following variables regressed out: percentage of mitochondrial counts, percentage of ribosomal counts, G2M and S phase scores, BCR genes’ expression scores. Selection of the number of components for the nearest-neighbor network computation was based on their visualization in an elbow plot. Features were clustered and cells visualized using UMAP (62).

BCR data were processed and mutational analysis performed using the Immcantation package (63). Clonal relationship was defined using the scRepertoire package with the “strict” method (64). BCR information was integrated with the scRNA-Seq data by merging the data with the metadata slot in the processed RNA-Seq Seurat object. Nebulosa plots were generated using the SCPubr package (65).

### ELISA.

IAV-specific antibody detection assay was performed on NW and sera from naïve and infected mice. Half-area flat bottom plates were coated the day before assay with 50 μL of a solution of the indicated antigen from PR8 at a concentration of 60 ng/mL and kept at 4 °C until use. Plates blocking was then performed with 2% FCS-supplemented PBS for an hour at room temperature (RT). Samples were diluted on the first row with a dilution factor of 4 and 50 for the NW and sera respectively and serially diluted with a factor 2. Plates were then incubated at 37 °C for 1.5 h before being washed 3× with 0.05% Tween PBS. 25 μL of a solution of anti-murine kappa-antibodies coupled with HRP was added in the wells for an hour at RT before being washed again. 25 μL of TMB solution was added to the well and left at RT for 5 min before stopping the reaction with the same volume of 2 M H_2_SO_4_.

### TCID_50_ Assay.

TCID_50_ assays were performed on nasal tissues and lungs after infection. Both types of organs were removed and weighed after being placed in ice-cold PBS for up to an hour. Nasal tissues and lungs were dissociated mechanically before centrifugation at 1,500×*g* for 7 min. The supernatants and their dilutions were used to infect MDCK cells and detect virus by lytic activity after 3 d at 37 °C. Method was based on ref. 66.

### Isolation of Cells from Murine Respiratory Organs.

Approximately 5 min before being killed, mice were injected intravenously by the tail with 3 μg anti-CD45 FITC (clone 53-2.1; BD Biosciences) to label leukocytes in the vasculature. Mice were then killed via isoflurane anesthesia followed by cervical dislocation and the respiratory organs were collected in PBS supplemented with 2% FCS. The tissues were placed into gentle-MACS C Tubes (Miltenyi Biotec) or Eppendorf tubes 1.5 mL with RPMI 1640 Medium (#22400089; Gibco) containing 30 μg/mL Collagenase (#05401127001; Roche) and 3 μg/mL DNAse (Cat. No. A7009; Sigma-Aldrich). The lung tissue was dissociated on the gentle-MACS Dissociator (Miltenyi Biotec) and then incubated at 37 °C for 45 min. The NT were collected in 1 mL of the digestion medium and incubated at 37 °C, 600 rpm for 45 min. Spleens, lungs, and NT were crushed on a 70 μm cell strainer cap and washed for 10 min at 400×*g*, 4 °C. Cell pellets were treated with red blood cell-lysis buffer 7 min for the NT and 9 min for Lungs and spleens. Cells were washed with FACS buffer and resuspended with the appropriate antibody cocktail. NALT were collected in FACS buffer and minced with scissors before being passed through filtered cap round-bottom FACS tubes and spun at 400×*g* for 5 min at 4 °C. Cell pellets were then resuspended in antibody cocktail.

### Spectral Flow Cytometry.

For murine samples: following collection of single-cell suspensions, cells were stained with NP and/or HA conjugated to Streptavidin-APC (Biolegend Cat#405243) or -PE (Thermo Fisher Cat#S866) and incubated at 4 °C in the dark for 2 h. Cells were then washed and incubated with the surface antibody cocktail on ice for 45 min. After two rounds of PBS washes, samples were incubated with Live Dead Fixable Far Red (1:1,000) in PBS at 4 °C for 20 min in the dark. All samples were then washed and fixed using 1.5%PFA solution and incubated at 4 °C for an additional 20 min. Acquisition followed the fixation. For surface staining, the following antibodies were used: CD19-BUV496 (eBio1D3 (1D3)), CD95-BUV737 (Jo2), CD69-BUV737 (H1.2F3), CD35-BV421 (8C12), IgM-BV421 or BV750 (RMM-1), CD38-BV785 (90), CD45-FITC (30-F11), IgA-TXRD (Polyclonal Southern-Biotech), CD138-Pe-Cy7 (281-2), CD38-APC-Cy7 (90), IgD-AF700 (11-26c.2a), B220-APC-Fire810 (RA3-6B2), CXCR5-BV421 (L138D7), PD-1-PE (29F.1A12), CD4-BV785 (RM4-5), CD86-FITC (GL1), CXCR4-ef450 (2B11).

For human samples: surface staining was proceeded in the same fashion as for the murine cells. Following Live/dead staining incubation, cells were washed and, when applicable, were fixed and permeabilized with eBioscience Foxp3/ Transcription Factor Staining Buffer Set (Invitrogen) according to manufacturer instructions. Intracellular transcription factor staining was performed in 1X Permeabilization Buffer (Invitrogen) at RT for 1 h before data acquisition by flow cytometry. For surface staining, the following antibodies were used: IgD-BUV395 (IA6-2), CD19-BUV496 (SJ25C1), IgM-BV605 (G20-127), CD20-BV785 (2H7), IgA-PercVio700 (IS11-SE10), CD38-AF700 (HIT2), CD10-APC (97C5), and IgG-FITC (G18-145). For intracellular transcription factor staining, the following antibodies were used: Bcl6 (clone: FJK-16s; Invitrogen).

All cells were acquired on the ID7000 (SONY) and analyzed using FlowJo 10.8.1 software (BD).

### Immuno-Histochemistry.

The upper part of the mice heads was collected, skinned, and enucleated before being placed in 1.5% Formaldehyde Solution (w/v) (ThermoScientific) solution at 4 °C for 24 h. The teeth and remaining muscle tissues were removed before transferring the head into fresh EDTA 0,5 M (Fischer) solution for a week at 4 °C. The head was cleaned again manually and embedded into sequential dilutions of OCT Paramount (Histolab) to be snap-frozen in isopropane using the liquid nitrogen cooling method. Samples were then either kept at −80 °C for long-term storage or sectioned. Frontal and sagittal sections were cut for a thickness of 10 to 12 μm and collected on SuperFrost microscopy slides and stored at −80 °C or were processed for staining. In the latter case, slides were baked for 1 h at 60 °C and fixed a second time with ice-cold acetone until the samples were dried. A PBS-based blocking solution containing 10% FBS and 5% Normal Rat Serum was applied to cover the samples at RT for 15 min. Samples were thereafter washed with PBS and stained with the antibody cocktail overnight at 4 °C in the dark. Hoechst 33342 solution (Thermo) was added on the slides for 15 min in the dark at RT, mounted with a fluorescent mounting medium (Dako) and left to dry for an hour before being acquired on an Axio Imager. Z2 (Zeiss) and analyzed on TissueFACS Analyzer (Version 7.1.120 Xylis).

For immuno-histochemistry staining, the following antibodies were used: B220-FITC/APC (RA3-6B2), GL7-AF687 (GL7), AID-biotinylated (mAID-2), Streptavidin-TXRD (Thermo Fisher Cat#S872), CD95-PE (Jo2), CD35-BV421 (8C12), FDC-M1 (FDC-M1) and Goat Anti-Rat Ig, Mouse ads-FITC (SouthernBiotech Cat. No.: 3010-02). Nuclei were stained using Hoechst 34580 (Thermo Fisher).

### Statistical Analysis.

Statistical analysis was performed by the unpaired *t* test and one-way ANOVA as indicated in the figure legends using Prism 10 (GraphPad Software). Graphs show mean ± SD.

### Study Approvals.

Mouse experiments were carried out under the ethical permits 1666/19, 3307/20, and 38/23 issued by the Swedish board of agriculture. Human experiments were approved by the Swedish Ethical Review Authority with Permit Nos. 2023-07055-01 and 2024-01433-01, with amendment 2024-06240-02. Written informed consent was obtained from either the patient or the legal guardians of the patient.

## Supplementary Material

Appendix 01 (PDF)

Dataset S01 (XLSX)

## Data Availability

Single-cell RNA sequencing raw and processed data (scRNA and BCR sequencing data) are deposited in the GEO database under Accession No. GSE289606 (67). All other data are included in the manuscript and/or supporting information.
