# Supplementary material for: Ectopic germinal centers in the nasal turbinates contribute to B cell immunity to intranasal viral infection and vaccination
Source: Proc Natl Acad Sci U S A. 2025 Mar 20;122(12):e2421724122. doi: 10.1073/pnas.2421724122 (PMC11962485; doi:10.1073/pnas.2421724122)
Supplement: Supplementary file 1 — Appendix 01 (PDF) [file pnas.2421724122.sapp.pdf]

Supporting Information for

Ectopic germinal centers in the nasal turbinates contribute to B cell immunity to intranasal viral infection and vaccination

Romain Gailleton[1], Nimitha R. Mathew[1], Laura Reusch[1], Karin Schön[1], Lydia Scharf [1], Anneli Stromberg[1,2], Andrea Cvjetkovic[3,4], Luaay Aziz[3,4], Johan Hellgren[3,4], Ka-Wei Tang[5,6], Mats Bemark[2,7], Davide Angeletti[1,8*]

Corresponding author: Davide Angeletti
Email: davide.angeletti@gu.se

This PDF file includes:

Figures S1 to S5

Other supporting materials for this manuscript include the following:

Dataset S1

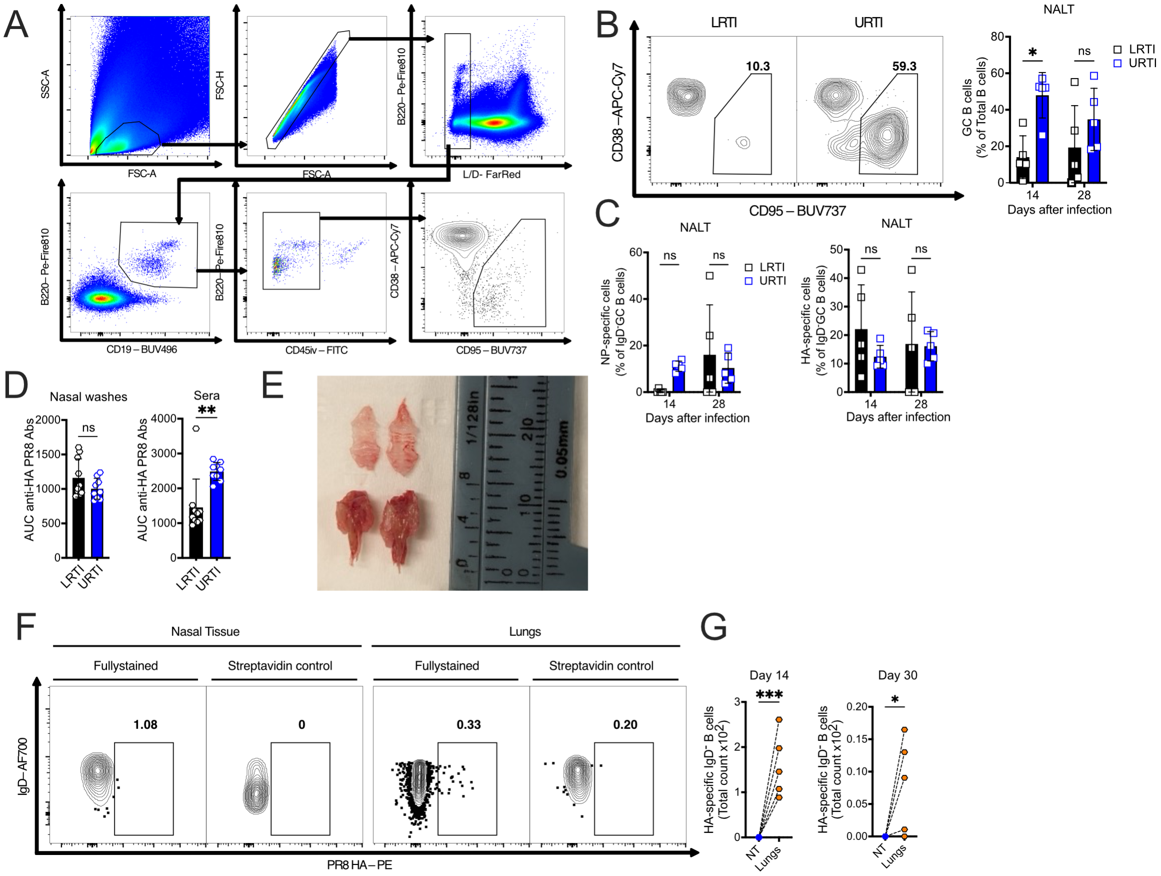

Fig. S1. **(A)** Gating strategy of NT GC B cells, example of a mouse NT at 21 days post URT infection with PR8. Arrows indicate the gating path. The strategy starts from the top left and terminates at the bottom right. **(B-C)** Bar graph comparison of total **(B)** or PR8-NP and PR8-HA specific **(C)** GC B cells percentages between URT and LRT infections at 14- and 28-days post infections in NALT. Linked representative flow plots of gated total GC populations are presented on the left of **(A)**. Mean ± SEM (n =5); one-way ANOVA test. **(D)** Bar graph comparison of calculated AUC from PR8-HA-specific Abs levels detected from nasal washes and sera of mice 28 days after URTI or LRTI. Mean ± SEM; (n=10) Student's t test. One representative experiment is shown from 2 independent experiments. **(E)** Picture of dissected NT (bottom) and NALT palate (top), centimetre graduated calliper for scale. **(F)** Representative flow cytometry plots of HA-specific IgD- B cells from nasal tissue and lungs, 14 days post intratracheal infection. Plots include fully stained and streptavidin control conditions. **(G)** Correlation dot plots showing the total count of HA-specific IgD$^-$ B cells in NT and lungs at 14 days (left) and 30 days post intratracheal infection (right). Mean ± SD. Statistical analysis performed using a t test (n=5). ns P >0.05; * P < 0.05; ** P < 0.005

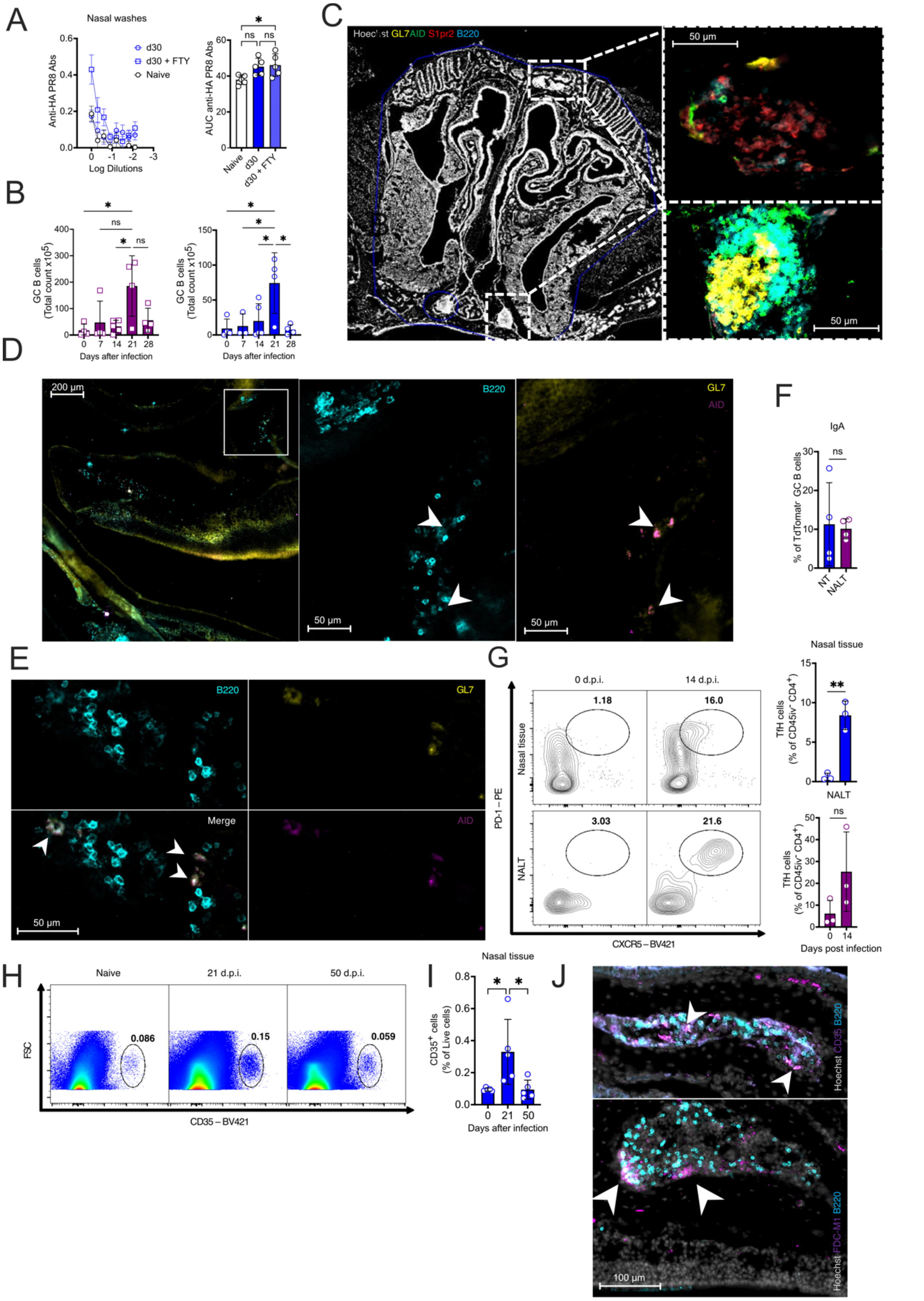

Fig S2. (A) Dilution curves of detected HA-specific Abs in NW from mice at steady-state or 28 days after URTI with or without FTY720 treatment. Mean ± SD; Bar graphs comparison of calculated AUC from above-mentioned groups. Mean ± SEM; one-way ANOVA test. One representative

experiment of 2 independent ones (n=5). **(B)** Bar graphs of HA-specific GC B cells total count in NT and NALT after URT infection. Mean ± SEM; (n=8-9), from 2 independent experiments; one-way ANOVA test. **(C)** Frontal section of a 21 d.p.i. URT infected mouse in overview (Left) and close-ups to DALT and NALT regions with indicated colours (Right). **(D-F)** NT microscopic section of a 21 d.p.i. URT infected mouse showing lymphoid structures at the lamina propria interface with the epithelium and the lumen and in the nasal turbinate showed in Fig. 2 G. The composite picture is shown on the left and the $GL7^{+}AID^{+}$ cells are shown on the right. **(E)** Bar graph comparison of IgA^{+} B cell proportions among TdTomato labelled $CD138^{-}IgD^{-}$ GC B cells at the 21 d.p.i. peak. Mean ± SEM; Student's t test. n=4-5. **(G)** Representative flow plots for follicular helper T cells in NT and NALT with comparative bar plots of TfH percentages among resident $CD4^{+}$ cells between 0- and 14-days post infection. Mean ± SEM; Student's t test (n=3). **(H)** Representative flow plots of NT $CD35^{+}$ cells at steady state or 21- and 50-days post URTI. **(I)** Bar graph group comparison of NT $CD35^{+}$ Live cells percentages from steady state or 21 and 50 days after URTI. Mean ± SEM; one-way ANOVA test. **(H-I)** One representative experiment of 2 independent (n=5). **(J)** Microscopic sagittal sections with indicated staining colours, close-up to nasal turbinate (in magenta: CD35 on top and FDC-M1 on bottom). ns $P > 0.05$; * $P < 0.05$; ** $P < 0.005$

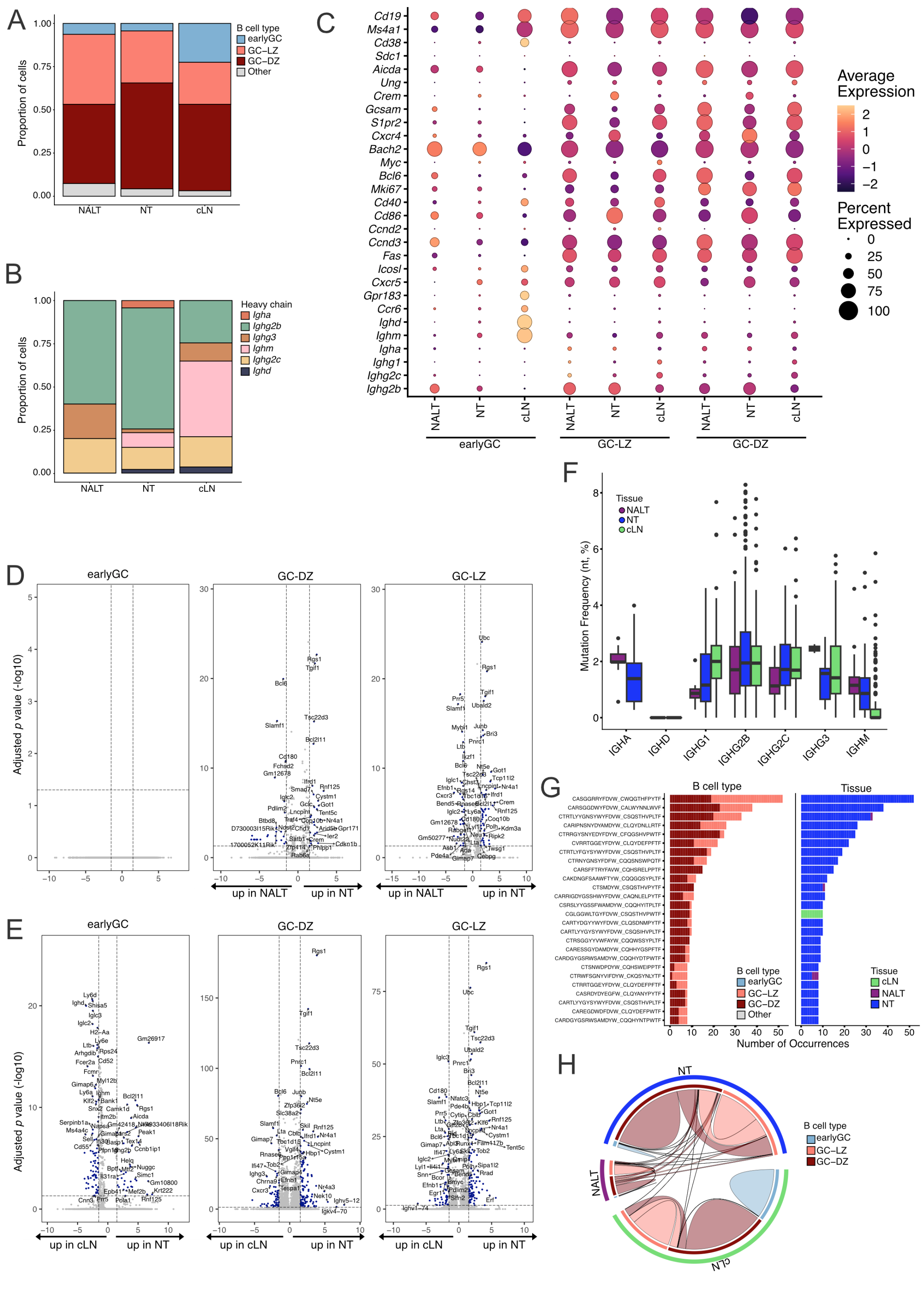

Fig S3. (A) Bar graph showing proportion of each UMAP cluster from Fig 3A, divided by tissue. **(B)** Bar graph showing proportion of each Ig heavy chain isotype, divided by tissue. **(C)** Dot plot representing mean expression of selected marker genes for each cluster and tissue. Color intensity

from blue to yellow indicates average expression of genes and size of the dot depicts percentage of cells expressing the gene within the clusters. **(D)** Volcano plot for differential expressed genes in the NT in comparison to the NALT for the three clusters identified in Fig 3A. The dotted lines indicate fold change and adjusted p value cutoffs. **(E)** same as in C but for NT in comparison to cLN**. (F)** Box plot showing the BCR nucleotide mutation frequency among B cell isotypes across tissues (NT, blue; NALT, violet; cLN, green). Data are presented as median and interquartile range. **(G)** Bar plot characterizing the top 26 most expanded clones. The y axis shows the CDR3 amino acid sequences of heavy and light chains while the bar graphs show the number of cells divided according to B cell cluster or tissue of origin. **(H)** Circos plot showing clonal relationship across tissues. Lines connecting across tissues indicate a shared clonotype. The section of the circle represents the relative frequency of the clone(s). Lines are colored according to B cell cluster as in Figure 3A.

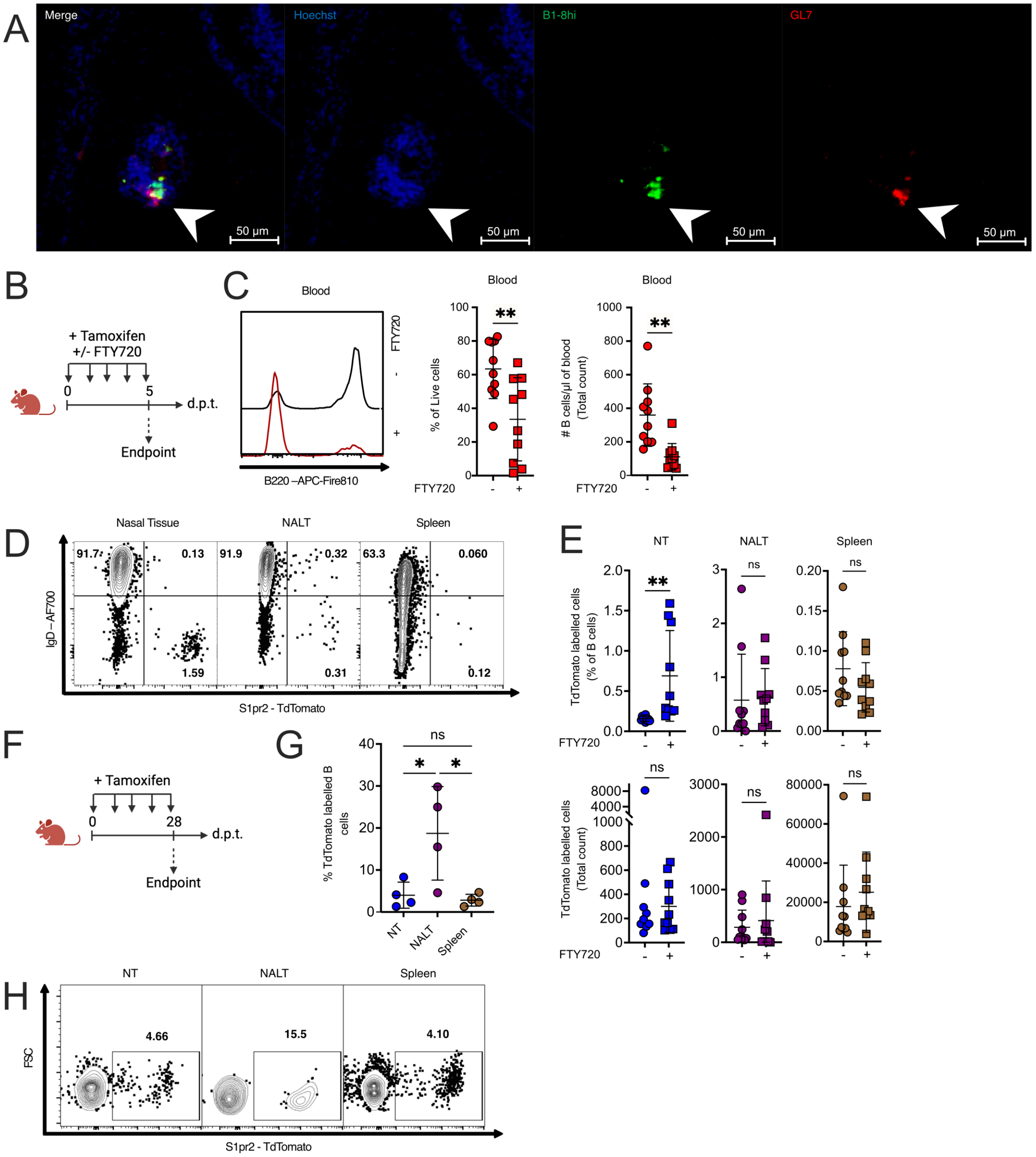

Fig S4. (A) BM-like structure observed in the NT of an NP-CT immunized mouse, showing Bi-8hi specific B cells co-expressing GL7**. (B)** Schematic representation of the experimental strategy for S1pr2 mice treated with tamoxifen, with or without fingolimod treatment for 5 consecutive days at steady state. Created with BioRender.com**. (C)** (Left) Histogram plot showing circulating live cells and B220 expression in fingolimod-treated and untreated mice. (Right) Dot plots showing the percentage and total count of B cells in the blood of treated and untreated mice. Data pooled from two independent experiments (n=10). Mean ± SD. Statistical analysis performed using a t test**. (D)** Representative flow cytometry plots of B cells in the NT, NALT, and spleen from mice shown in Supplementary Figure 3B**. (E)** Dot plots showing the percentage (top) and total count (bottom) of TdTomato-labelled B cells in the NT, NALT, and spleen of fingolimod-treated and untreated mice. Data pooled from two independent experiments (n=10). Mean ± SD. Statistical analysis performed using a t test**. (F)** Schematic representation of the experimental strategy for S1pr2 mice treated

with tamoxifen for 28 days at steady state. Created with BioRender.com. **(G)** Bar graph comparison of the percentages of TdTomato-labelled B cells from $CD45iv^-$ B cells in NT, NALT, and spleen, 30 days after the experiment start. Mean ± SEM (n=5). Statistical analysis performed using a one-way ANOVA test. **(H)** Flow cytometry gating of TdTomato-labelled B cells between these organs. ns P >0.05; * P < 0.05; ** P < 0.005; *** P < 0.0005; **** P < 0.00005

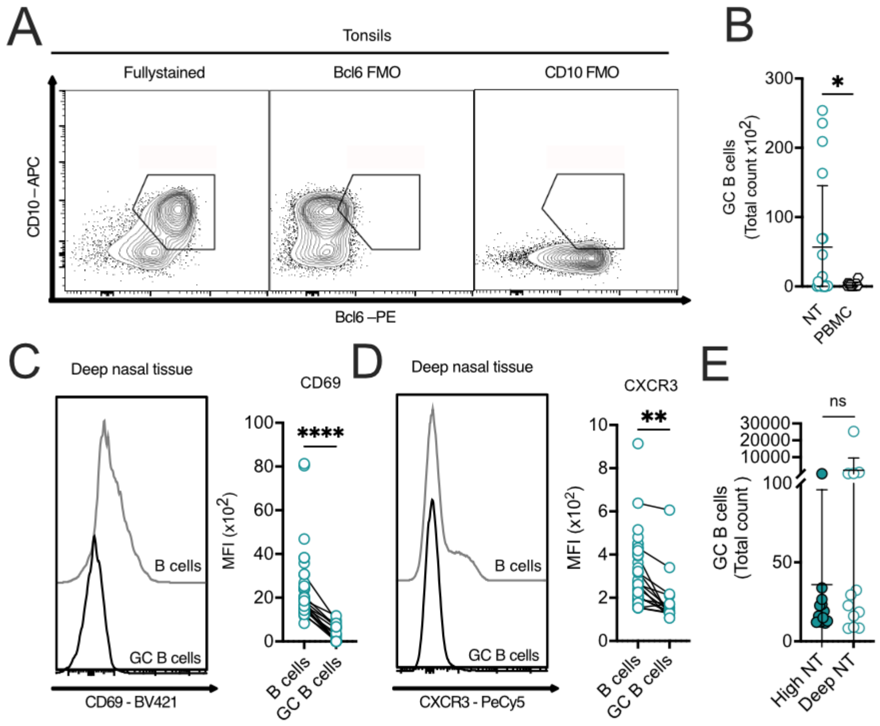

Fig S5. (A) Representative flow cytometry plots of human tonsil IgD$^-$ B cells showing detected expression levels of CD10 and Bcl6 in fully stained samples, as well as CD10 fluorescence-minus-one (FMO) and Bcl6 FMO controls. **(B)** Dot plot showing GC B cell count in NT and PBMC. Statistical analysis performed using a t test (n=36). Mean ± SD. **(C)** (Left) Histogram plot showing mean fluorescence intensity (MFI) of CD69 in B cells and GC B cells from deep human nasal tissues. (Right) Correlative dot plot quantification of CD69 expression. Statistical analysis performed using a t test (n=36). **(D)** (Left) Histogram plot showing MFI of CXCR3 in B cells and GC B cells from deep human nasal tissues. (Right) Correlative dot plot quantification of CXCR3 expression. Statistical analysis performed using a t test (n=36). **(E)** Dot plot showing GC B cell count in Deep and High NT. Statistical analysis performed using a t test (n=12). Mean ± SD. ns P >0.05; * P < 0.05; ** P < 0.005; *** P < 0.0005; **** P < 0.00005

Dataset S1 (separate file). Differentially expressed genes across different clusters for nasal tissue *vs* cLN and nasal tissue *vs* NALT
